# Tiletamine-Zolazepam, Ketamine, and Xylazine Anesthetic Protocol for High-Quality, High-Volume Spay and Neuter of Free-Roaming Cats in Seoul, Korea

**DOI:** 10.3390/ani14040656

**Published:** 2024-02-19

**Authors:** Donghwi Shin, Yoonju Cho, Inhyung Lee

**Affiliations:** 1 Department of Veterinary Clinical Sciences, College of Veterinary Medicine and Research Institute for Veterinary Science, Seoul National University, Seoul 08826, Republic of Korea; hwi4010@snu.ac.kr; 2 Research Institute, VIP Animal Medical Center, Seoul 02830, Republic of Korea

**Keywords:** anesthesia safety, autonomic stability, castration, free-roaming cat, injectable anesthesia, ovariohysterectomy, population control, TNR

## Abstract

**Simple Summary:**

Surgical anesthetic management during short-term procedures such as sterilization is highly relevant due to the hemodynamic, cardiorespiratory, and autonomic alterations that could be present. In the case of sterilization aimed at free-roaming cats, a drug combination to provide hypnosis, analgesia, and autonomic balance can reduce these anesthetic risks. Cat neutering through trap-neuter-return (TNR) programs is a non-lethal alternative for free-roaming cat population control. This study is an evaluation of anesthesia used in the high-quality, high-volume spay and neuter (HQHVSN) model of a TNR program for free-roaming cats in Seoul, Korea. A combination of tiletamine-zolazepam, ketamine, and xylazine (ZKX) was intramuscularly administered to obtain anesthesia. The evaluation was based on the records of 1261 cats with complete records of the injected volume of anesthetics and times, out of a total of 1361 cats. The study confirmed the safety and efficacy of the ZKX combination administered IM in a TNR program in the HQHVSN model and provided a range of appropriate doses. This will enable TNR programs to be more effective and contribute to a stable free-roaming cat population that can be successfully controlled for welfare.

**Abstract:**

This study was performed to evaluate the anesthetic protocol used in the high-quality, high-volume spay and neuter (HQHVSN) of free-roaming cats in Seoul, Korea from 2017 to 2022. The evaluation was performed on a total of 1261 free-roaming cats, with an average weight of 3.48 ± 1.04 kg. The anesthetic combination tiletamine-zolazepam, ketamine, and xylazine (ZKX) was injected intramuscularly. The actual drug doses administered were tiletamine-zolazepam 5.52 ± 1.70 mg/kg, ketamine 8.94 ± 3.60 mg/kg, and xylazine 1.11 ± 0.34 mg/kg. Additional doses were required in 275 cats out of a total of 1261 (21.8%). Following anesthesia and surgery, 1257 cats (99.7%) were returned to their original locations. Four cats (0.3%) died postoperatively. The mean duration of anesthesia (from ZKX combination to yohimbine administration) was 26 ± 22 min for males and 55 ± 36 min for females, while the time from yohimbine administration to the recovery was 31 ± 22 min for males and 20 ± 17 min for females. The use of ZKX for HQHVSN of free-roaming cats is inexpensive, provides predictable results, can be administered quickly and easily in a small volume, and is associated with a low mortality rate during the first 72 h post-surgery.

## 1. Introduction

Buildings constructed for human habitation such as apartments, shopping districts, schools, and houses in South Korea often host cats referred to as ‘street cats’. These cats primarily survive by preying on mice and rats or scavenging through discarded waste in the vicinity. These free-roaming cats (FRCs) are also termed as ‘free-living’, ‘stray’, ‘community’, or ‘colony’ cats [1,2,3]. Since 2008, South Korea has implemented trap-neuter-return (TNR) programs, releasing cats back to their capture locations after spaying or neutering surgeries, as a means to control the population of FRCs. From 2010 to 2022, approximately 120,000 FRCs in Seoul were subjected to TNR according to data aggregation [4]. The neutering procedures through TNR serve as a non-lethal alternative for population management. Several studies in Australia, Israel, New Zealand, the USA, and elsewhere have highlighted the effectiveness of TNR in controlling cat populations and reducing issues such as euthanasia associated with cat overpopulation over time [2,5,6,7,8,9]. Moreover, the aggressive tendencies in male cats can be reduced through such programs, leading to a decline in noise-related issues [7]. Additionally, targeted TNR initiatives have proven more efficient in controlling FRC populations compared to sporadic TNR efforts [5,10]. According to a 2021 research report, Population Monitoring and Management Strategy of the free-roaming cats in Seoul, these efforts have reduced the number of FRCs in Seoul from a high of 203,615 in 2015 to about 90,000 in 2021. In 2021, it was also estimated that about 50% of the FRCs in Seoul were neutered, based on observations of ear-tipped cats. Consequently, many countries are refining and advancing TNR approaches tailored to areas with high cat populations, striving to expand and enhance these efforts [6,9,11,12,13].

Seoul Metropolitan City has been piloting and operating the Operation Catnip program, a highly effective trap-neuter-return method implemented by the University of Florida since 2017, in order to enhance efficiency. The application and outcomes of this high-quality, high-volume spay and neuter (HQHVSN) approach have been documented in another study [1]. South Korea supports TNR initiatives when citizens apply for FRC neutering surgeries through local governments. Since the support is allocated from both national and local budgets, addressing complaints equitably necessitates sporadic TNR activities. Therefore, Seoul aims to expand its strategy, led by local authorities, targeting specific areas intensively for TNR. Conducting large-scale neutering surgeries for FRCs requires significant personnel resources due to the need to neuter a large number of cats at once. Establishing a comprehensive process, especially to minimize mortality rates, becomes crucial in this endeavor. Among various considerations, developing and implementing a straightforward yet safe anesthetic protocol for neutering surgeries is essential and arguably the most crucial aspect. For an optimal anesthetic protocol for FRC neutering, it is preferable to utilize drugs that are safe, offer rapid onset and predictable anesthesia, provide postoperative analgesic effects, are reversible, and are cost-effective. Among the drugs used for injectable anesthesia, tiletamine-zolazepam, ketamine, and xylazine meet these criteria to some extent. Therefore, combinations of these drugs are employed in many large-scale FRC neutering programs [14,15].

In a large-scale FRC neutering program ongoing in Seoul for several years, the tiletamine-zolazepam, ketamine, and xylazine (ZKX) combination has been employed. This research seeks to assess the effectiveness of the ZKX combination in providing sufficient anesthesia for neutering procedures. The study also aims to evaluate the dosages of each drug administered based on the actual body weight of the cats and the resultant anesthetic effects. The hypothesis posits that the ZKX combination will offer reliable and humane anesthesia for FRC neutering. The outcomes of this investigation are expected to contribute significantly to the optimization of TNR programs not only in Seoul but also in diverse regions across South Korea and globally.

## 2. Materials and Methods

Seoul, divided into 25 administrative districts, has an FRC guardian association in each autonomous district. The Seoul Metropolitan Government collaborates with these associations to discuss FRC management. Regions that agree to capture and release cats for neutering are included in the Seoul TNR program, where the operations are conducted. From 2017 to 2022, the HQHVSN system TNR program has been implemented multiple times. Due to the absence of dedicated facilities for HQHVSN in South Korea, makeshift clinics were established in vacant buildings owned by both the Seoul Metropolitan Government and Gyeonggi Province to execute the project. Gyeonggi Province included captured FRCs from two redevelopment areas in 2020 and 2021. Since the same TNR protocol was applied in this study, the anesthesia and surgical procedures for all cats were the same regardless of the region. Caregivers, residents of each autonomous district acting as FRC guardian associations, helped with the trapping, post-operative management, and return of FRCs. The purpose of the project, methods of trapping and return, and process of anesthesia and surgery were explained to them in advance. Caregivers captured FRCs using either manual or automatic trap cages. Before surgery, the cats rested and fasted for 8 h in holding areas. Upon arrival at the makeshift clinic, and transported through an individual trap cage, each cat was assigned an identification number and moved through a series of treatment stations after anesthesia before being transferred to the surgical room. Before administering anesthesia, veterinarians assessed the overall health and appearance of the cats’ wound or bleeding to ensure each individual’s suitability for anesthesia. FRCs that were markedly underweight for their body size or showed signs of respiratory distress were not administered anesthetic drugs and were excluded. Those with severe physical trauma or suspected chronic infectious diseases were also excluded from the neutering program.

The anesthetic consisted of a blend of tiletamine-zolazepam powder (250 mg, Zoletil^®^50, Virbac Korea Co., Ltd., Seoul, Republic of Korea), 6 mL of ketamine (50 mg/mL, Yuhan Ketamine 50 Inj., Yuhan Corporation, Seoul, Republic of Korea), and 0.5 mL of xylazine (100 mg/mL, Xyzine-plus Inj., ESEF Co., Ltd., Ansan, Republic of Korea). This combined anesthetic solution contains 38.4 mg of tiletamine-zolazepam, 46.2 mg of ketamine, and 7.7 mg of xylazine per 1 mL. The trap cage was positioned upright, and a divider was placed inside to restrict the cat’s movement. The ZKX combination was administered into the back muscles (erector spinae) or femoral muscles (biceps femoris or quadriceps femoris, depending on the situation). Body weight was estimated using a visual body condition scale based on the cat body condition score table updated in 2020 by the World Small Animal Veterinary Association (WSAVA) Global Nutrition Committee [16], and the ZKX dosage was determined as 0.15 mL per kg of ideal body weight. Upon confirming sufficient anesthesia induction (unconsciousness and muscle relaxation) after a few minutes, the cats were released from the trap cage. Subsequently, the body weight, heart rate (HR), respiratory rate (RR), body temperature (BT), and mucous membrane color (MMC) of the cats were assessed and recorded. All cats were administered both prophylactic antibiotics, consisting of penicillin G + dihydrostreptomycin sulfate (25,000 IU/kg, PPS, Daesung Microbiological Labs. Co., Ltd., Uiwang, Republic of Korea), and meloxicam (0.3 mg/kg, Metacam, Boehringer Ingelheim Korea Ltd., Seoul, Republic of Korea) for pain management before surgery. The hair on the surgical site was clipped, and the cats were transferred to the operating room and positioned in dorsal recumbency. They were placed on bubble cushion wrap or a heating mat to maintain BT, and the surgical site was disinfected with a 4% chlorhexidine solution, diluted from a 5% solution (Alpha Hexidine 5% solution, Firson Co., Ltd., Cheonan, Republic of Korea). For females, a midline incision was made for ovariohysterectomy. For males, the scrotal approach was made for orchiectomy. The surgeries were performed following appropriate methods for HQHVSN, such as pedicle tie and modified Miller’s knot [17]. Although there are no records, the assistants of each operation table were instructed to check the HR and RR at least every five minutes during the surgery. In case of inadequate oxygenation, such as apnea, preparations were made to manually supply oxygen through endotracheal intubation. If the cats showed insufficient anesthetic level or exhibited movement during surgery, additional doses of ZKX were administered. The dose and number of injections were determined by the remaining time of surgery and the anesthetic levels of the cat. Additional injections of anesthetics were administered up to three times into the biceps femoris or quadriceps femoris.

Post-surgery vaccinations included the FVR-CP vaccine (Nobivac^®^ Tricat Trio, MSD Animal Health Korea Ltd., Seoul, Republic of Korea) and the Rabies vaccine (Nobivac^®^ Rabies, MSD Animal Health Korea Ltd., Seoul, Republic of Korea) in the left and right distal limbs, respectively. Topical deworming agent (Advocate^®^, Bayer Korea Ltd., Seoul, Republic of Korea) was applied to the skin at the base of the skull after surgery. A subcutaneous injection of 0.9% normal saline (30–50 mL, depending on weight, Isotonic Sodium Chloride Injection 100 mL, Dai Han Pharm. Co., Ltd., Seoul, Republic of Korea) was administered interscapularly for all cats after surgery [18]. The BT of each cat was checked, and yohimbine (0.1 mg/kg, Zyverse Inj., ESEF Co., Ltd., Ansan, Republic of Korea) was intravenously administered through the median saphenous vein. Each cat was monitored until recovery, which refers to the state of being conscious and normal righting reflex.

The duration of anesthesia was defined as the time from the administration of the ZKX combination to the administration of yohimbine. Following yohimbine administration, each FRC was monitored until recovery; however, due to limited personnel resources, records of recovery duration for all cats were unattainable. After the recovery, FRCs stayed in a separate quiet space adjacent to the surgical room and received food and water from caregivers. Throughout the research period, various brands of cat food were provided, and a mixture of cat-specific dry food and wet food was given according to the recommended amounts by the food companies. The cats were monitored continuously to respond to any emergencies; males were observed for 24 h, and females for 72 h. The caregivers observed whether there were any blood or discharge on the absorbent pads placed under the traps during this period. The veterinarian confirmed the surgical site of FRCs outside the trap before they were released back to their original capture locations. The surgery and all of the invasive procedures were only performed by certified veterinarians.

Comparative analysis of the data about total injected volume between male and female groups, and about body weight between single and multiple injected groups were performed using IBM SPSS Statistics (V26). The Shapiro-Wilk test was performed to test the data’s normality distribution, and the Mann-Whitney test was performed to compare certain parameters.

## 3. Results

Throughout the six-year study, multiple TNR programs were implemented, encompassing a comprehensive dataset of 1261 cats distributed across the years as follows: 150, 224, 88, 358, 224, and 217 cats, respectively. This number comprised 636 intact females (50.4%) and 625 intact males (49.6%). A total of 1257 cats (99.7%) that recovered successfully after surgery and were confirmed to have no problems during the 24 h for males and 72 h for females were released back to their initial capture sites, whereas four female cats (0.3%) did not survive during the programs. Three of these cats were pregnant, the other was nursing.

The vital parameters were assessed immediately after induction of anesthesia. The mean ± standard deviation HR was 152.5 ± 37.4 beats per minute, RR was 41.5 ± 19.8 breaths per minute, and BT was 38.9 ± 3.0 °C. A pink MMC was confirmed on most cats, and a pale pink color was found on 123 cats out of 1261 (9.8%).

The average body weight of the presented cats was 3.48 ± 1.04 kg, with males weighing about 0.6 kg more than females. The median and interquartile range of the total injected ZKX volume were 0.50 and 0.20 mL for males and 0.45 and 0.10 mL for females, respectively. The mean total volume of ZKX administered to males was significantly higher than females (*p* = 0.02) (Table 1).

For males that underwent surgery with a single injection, the administered ZKX volume averaged 0.47 ± 0.13 mL. In contrast, females received an average ZKX dosage of 0.45 ± 0.10 mL. The ZKX dosage per kg was 0.01 mL/kg higher in females than in males. Among males requiring additional anesthesia (123 out of 625, 19.7%), their total ZKX dosage averaged 0.66 ± 0.22 mL. The dosage per kg for this subgroup was 0.16 ± 0.05 mL/kg. For females (152 out of 636, 23.9%) necessitating supplemental anesthesia, their average ZKX dosage was 0.61 ± 0.17 mL. The per kg dosage for this subgroup was 0.19 ± 0.05 mL/kg. The median and interquartile range of the body weight in the single injected group were 3.33 and 1.27 kg, respectively, while for the multiple injected group, the data were 3.55 and 1.40 kg, respectively. Significant differences between the body weight of the single and multiple injected groups were confirmed (*p* = 0.000141). The median and interquartile range of the body weight in the single injected male group were 3.70 and 1.60 kg, respectively, while for the multiple injected group, the data were 4.37 and 1.50 kg, respectively. Significant differences between the body weight of the single and multiple injected male groups were confirmed (*p* = 0.000004) (Table 2).

The duration of anesthesia was 26 ± 22 min for males and 55 ± 36 min for females. A total of 593 cases had measured time to recovery from yohimbine administration. The average time of 284 males was 31 ± 22 min, and 309 females was 20 ± 17 min.

## 4. Discussion

Ensuring consistent and appropriate anesthesia throughout the neutering surgery is crucial, necessitating an anesthetic protocol for FRCs that not only swiftly induces unconsciousness but also muscle relaxation, amnesia, inhibition of reflexes, and, especially, analgesia to minimize stress and expedite the procedure [19]. Additionally, the drug used should have a broad safety margin, and, if possible, have an antagonist. Given the considerations for safety, efficiency, and time constraints in TNR programs, which utilize intramuscular injections for anesthesia, it is imperative to employ drugs amenable to injectable anesthesia. The ZKX utilized in this study comprises tiletamine-zolazepam, ketamine, and xylazine. Zolazepam belongs to the benzodiazepine class, specifically a diazepinone minor tranquillizer. It possesses muscle relaxant and anticonvulsant properties. Tiletamine, on the other hand, is a phencyclidine derivative cyclohexane drug, similar to ketamine, acting as a dissociative agent. When administered alone, it can induce a cataleptic, dissociative state similar to that of ketamine. At higher doses, especially in cats, it can lead to unconsciousness and a surgically anesthetic state. However, to counteract potential side effects such as seizures or muscle rigidity, it is co-administered with zolazepam for synergistic effects. The combination of tiletamine and zolazepam, when administered intramuscularly to cats, has an onset of action between 1–7 min, inducing either sedation or general anesthesia depending on the dosage, and the duration of action is around 30–60 min. Depending on the dose, it has a longer duration of action than ketamine [20], so it may provide some benefit for post-operative analgesia, as the analgesic effect lasts long after the patient regains consciousness. Ketamine is also a phencyclidine derivative drug, inducing a cataleptic, dissociative anesthetic state similar to that induced by tiletamine. Ketamine acts as a non-competitive antagonist at the N-methyl-D-aspartate (NMDA) glutamate receptor. Additionally, it interacts with various other binding sites, including non-NMDA glutamate receptors, nicotinic and muscarinic cholinergic receptors, and opioid receptors, thereby exhibiting analgesic effects. When administered intramuscularly, its maximum anesthetic effect is observed around 10 min from the injection. While effective for somatic pain control, it has a relatively short duration of approximately 30 min. Both tiletamine and ketamine have low pH levels (approximately 2.0–3.5 and 3.5–5.5, respectively), which can induce pain upon intramuscular injection [20]. Xylazine acts as an α_2_ adrenergic receptor agonist, producing sedative and visceral analgesic effects through central nervous system (CNS) depression. Additionally, it induces muscle relaxation [21]. The analgesic effects are not dose-dependent and are suitable for surgeries lasting up to 30 min due to relatively short duration. Furthermore, reversal of the drug effects is possible through the administration of an α_2_ adrenergic receptor antagonist, such as yohimbine. The reason for using xylazine as an α_2_ adrenergic agonist in this study is because it offers the advantages of reduced injection volume and economic benefits. Xylazine at a dose of 1.1 mg/kg was assumed to be equivalent to medetomidine at 40 µg/kg [22]. This calculation confirmed that the amount injected into cats was approximately 4 times less when using 100 mg/mL xylazine compared to commercially available 1 mg/mL medetomidine.

A combination of drugs with the aforementioned characteristics allows for reduced individual drug quantities, thereby minimizing the volume of each drug while targeting multiple receptors in the CNS to induce both analgesia and unconsciousness. The pharmacological characteristics of the drugs within ZKX have been examined in previous studies [14,23]. The ketamine in the ZKX anesthetic mixture offers pre-emptive visceral analgesia, mitigating surgery-related noxious stimuli’s impact on central nervous system (CNS) sensitivity [24]. It is complementary to tiletamine-zolazepam, which can provide a longer analgesic effect than ketamine. By substituting water with ketamine and xylazine in the tiletamine-zolazepam mixture, a balanced anesthetic is achieved, targeting different CNS receptors, and reducing individual drug dosages while enhancing analgesia and sedation [14]. A small-volume muscle injection allows for easy administration and provides sufficient unconsciousness and both somatic and visceral analgesia for short surgical procedures lasting approximately 30 min, without significantly affecting spontaneous respiration [20]. The suppression effect on the cardiovascular system is minimal or even stimulative, which offers advantages in maintaining blood pressure and makes it a safe combination of drugs with a broad safety margin. It also has a relatively short recovery time, and the residual effects of xylazine can be reversed with yohimbine if needed [14]. In South Korea, where it is difficult to use high-potency opioids in unowned animals, this combination was convenient to use in a large-scale TNR program for spaying and neutering cats.

Using a small amount of anesthetic allows for rapid administration and minimizes stress for the cat. U.S. non-profit organization Operation Catnip^®^ mixed 6 mL of ketamine (100 mg/mL) and 1 mL of xylazine (100 mg/mL) in a bottle containing zolazepam 250 mg and tiletamine 250 mg. The recommended injection volume was 0.25 mL, administering zolazepam at 8.9 mg, tiletamine at 8.9 mg, ketamine at 21.4 mg, and xylazine at 3.6 mg for each cat. One study adapted the protocol used in Operation Catnip^®^. The researchers mixed in a bottle containing zolazepam 250 mg and tiletamine 250 mg, with 4 mL of ketamine (100 mg/mL) and 1 mL of xylazine (100 mg/mL) [14]. The approximate weight of the FRC inside the trap cage was estimated to be 3 kg, designed to administer 0.25 mL each. It contains 12.5 mg of zolazepam, 12.5 mg of tiletamine, 20 mg of ketamine, and 5 mg of xylazine. In South Korea, however, it is not possible to obtain high-dosage tiletamine-zolazepam and ketamine products. Therefore, a different anesthetic formulation, containing 125 mg each of zolazepam and tiletamine, was used. Additionally, a 100 mg/mL ketamine product was unavailable in South Korea, so a 50 mg/mL product was used. The anesthetic was prepared according to the mixing ratio of Operation Catnip^®^, containing 19.2 mg/mL of each zolazepam and tiletamine, 46.2 mg/mL of ketamine, and 7.7 mg/mL of xylazine. The veterinarian administering the anesthetic estimated the weight of the cat in the trap cage and administered 0.15 mL per kg, which contains 17.2 mg, 20.8 mg, and 3.5 mg of tiletamine-zolazepam, ketamine, and xylazine, respectively, based on 3 kg. These doses produced sufficient unconsciousness, muscle relaxation, and analgesia to spay and neuter the FRCs, and appeared to have a large safety range. Also, although the injection volume increased compared to using high-dosage anesthetics, due to the small absolute volume, there were no difficulties in administering intramuscular injection on-site. Moreover, with drugs that contain high dosages in a small volume, even a small change in injection volume can have a critical impact, so using slightly diluted dosages increases the safety margin of the anesthetic [25].

The most significant advantage of the anesthetic combination used in this study can be identified as its broad safety margin. While administering anesthesia based on an accurate assessment of an individual’s weight was the assumed safest method, situations arise in the treatment of FRC where precise weight measurement was unavailable, necessitating anesthesia without such data. In this context, ZKX can mitigate concerns regarding the risks associated with estimating FRC’s weight for injection. Moreover, even if there are concerns about anesthetic accidents due to overdose, there is the advantage of potentially expecting primary arousal through the administration of yohimbine, which can reverse the effects of xylazine. A study that conducted a large-scale survey of feline anesthesia retrospectively reported a mortality rate of 0.3% (35/11,227) [26]. According to the survey results, most respondents said they did not measure the weight (82%) or sedate the cats before anesthesia (74%). In addition, more than 50–90% of the cats (median 77%) were identified as young and healthy, and most of the anesthesia proceeded without intubation (93%). It is similar to the results of the current study, which was conducted with injectable anesthesia using estimated body weight without sedation. This 2017–2022 HQHVSN project, which was conducted intermittently by veterinarians who wanted to volunteer at makeshift clinics, also had a mortality rate of 0.3% (4/1261). The unexpected fatalities were pregnant or nursing. Post-mortem diagnoses were not conducted, and the precise causes of death remained undetermined. However, these cats were suspected to have experienced hemorrhage and a significant reduction in BT due to prolonged surgical duration and the development of mammary glands. To avert potentially fatal complications, facilities should be adequately equipped for anesthesia administration, patient monitoring, and emergency response [27].

In cases where the average weight of males was 3.69 ± 1.17 kg and females were 3.14 ± 0.78 kg, administering 0.14 ± 0.04 mL/kg for males and 0.15 ± 0.04 mL/kg for females maintained a stable anesthetic state. However, when slightly lower doses of 0.12 ± 0.03 mL/kg for males and 0.13 ± 0.03 mL/kg for females were administered, additional anesthesia was required. Significant differences in the total injected volume of the ZKX combination were confirmed between males and females. No significant differences were identified between multiple injected males and females, suggesting that this is likely to be simply due to insufficient dosage at the first drug injection. Therefore, it seems more appropriate to refer to the whole sample. However, Significant differences in the body weight between single and multiple injected groups were confirmed. Based on these findings, administering 0.15 mL per 1 kg of a cat’s weight is proposed. As the group requiring multiple injections had a significantly higher body weight, to reduce additional injections, care should be taken to avoid underdosing in individuals with presumed higher body weight. However, obesity increases the risk of anesthetic accidents, so it is essential to limit increasing the anesthetic dose in proportion to the cat’s weight alone. Cats weighing more than 6 kg are known to have a three times higher risk of death during the perioperative period compared to cats weighing between 2–6 kg [28,29]. Therefore, for overweight cats, increasing the dose from the standard 0.5 mL is recommended but ensuring it remains within the 0.9 mL limit for a 6 kg cat.

This study has some limitations which are a challenge for further research that should be addressed. First, efforts were made to prioritize patient safety within the given conditions; however, the system for monitoring and recording the condition of FRCs before and after anesthesia and surgery was insufficient. Having equipment for patient monitoring would be beneficial, but it is not essential to have an expensive veterinary multiparameter monitor. Various alternative methods can be employed to comprehensively evaluate the overall condition and recording. Second, the program requires more reliable pain management. Furthermore, more reliable pain management is required. Effectively addressing a cat’s pain after neutering is also important, given that cats tend not to display it unless experiencing significant discomfort. While the veterinarians administered meloxicam (Metacam) at 0.3 mg/kg for subcutaneous injection, studies suggest that enhancing analgesia can be achieved through local anesthesia with lidocaine or bupivacaine [30,31,32]. Administering bupivacaine 0.25% at 2 mg/kg for incision line injection and intraperitoneal administration in cats undergoing ovariohysterectomy could provide adequate post-operative pain relief, reducing stress during anesthetic recovery and aiding in the cat’s recovery. For males, it is recommended to inject 2% lidocaine at 1–2 mg/kg into the testicle. While both bupivacaine and ropivacaine injections are effective, lidocaine is preferred due to the high vascular distribution of bupivacaine and ropivacaine within the testicles, which could pose cardiac toxicity risks [33,34]. Additionally, localized anesthesia can reduce the frequency of additional anesthesia and potentially lower the dosage required, which might be considered in future anesthetic processes. Lastly, it is essential to determine the cause of death through postmortem examinations. Identifying areas for improvement through this process and implementing necessary changes are crucial for advancement. However, due to the cultural atmosphere in South Korea and the characteristics of FRC neutering programs, conducting postmortem examinations on deceased cats is challenging. However, to align efforts with the goals of both the neutering programs and the welfare of FRCs, collective endeavors should be made toward the common objective. Increased attention and assistance from volunteers including veterinarians are required for this purpose. Also, education for veterinarians, operation table assistants, and caregivers is necessary.

## 5. Conclusions

South Korea primarily implements TNR in animal hospitals under local government consignment contracts. The types and amounts of anesthetic agents may vary depending on the individual veterinarian’s anesthetic protocol. However, for planning and operating high-volume spay and neuter procedures, standardized procedures are necessary. Especially applying a stable anesthetic protocol is crucial not only for the success of the HQHVSN program but also for instilling trust in the surgeons who administer it. This study evaluates the dosage and effects of the ZKX combination, employed in the HQHVSN project conducted in Seoul, South Korea, between 2017 and 2022. Through this evaluation, it was determined that the ZKX combination offers effective anesthesia for FRC neutering surgeries. Due to the wide margin of safety of ZKX anesthesia, it can ensure safe anesthesia even in the environment of TNR surgeries where accurate measurement of body weight is not feasible. The appropriate combination of four drugs induces effective and predictable unconsciousness and analgesic effects in cats, reducing their stress due to rapid administration via intramuscular injection in minimal volumes. Additionally, the inclusion of antagonist for specific drug allows for the use when necessary. Various local governments plan to expand public facilities for the neutering of FRCs, aiming to capture and safely neuter a greater number of them. The results of this study suggest that the establishment of a standardized anesthetic protocol will contribute to the execution of high-quality TNR programs, and it can be used as a basis for the FRC population management policy.

## Figures and Tables

**Table 1:** Actual body weight and dosage of anesthetics according to actual body weight of cats.

Sex (N)	Body Weight (kg)	Total Volume (mL)	Zolazepam (mg/kg)	Tiletamine (mg/kg)	Ketamine (mg/kg)	Xylazine (mg/kg)
Total (1261)	3.48 ± 1.04	0.50 ± 0.15	2.76 ± 0.85	2.76 ± 0.85	8.94 ± 3.6	1.11 ± 0.34
Male (625)	3.79 ± 1.17	0.51 ± 0.17 *	2.59 ± 0.84	2.59 ± 0.84	8.73 ± 3.74	1.03 ± 0.34
Female (636)	3.18 ± 0.77	0.49 ± 0.14	2.96 ± 0.84	2.96 ± 0.84	9.31 ± 3.49	1.19 ± 0.34

Values are presented as mean ± SD. * Significant difference between male and female group (*p* = 0.02).

**Table 2:** Volume of anesthetics according to the number of injection and actual body weight of cats.

Groups	Sex (N)	Body Weight (kg)	1st Volume (mL/kg)	Additional Volume (mL/kg)
Single dose	Total (986)	3.42 ± 1.03 *	0.14 ± 0.04	-
	Male (502)	3.69 ± 1.17 *	0.14 ± 0.04	-
	Female (484)	3.14 ± 0.78	0.15 ± 0.04	-
Multiple doses	Total (275)	3.69 ± 1.03	0.13 ± 0.03	0.06 ± 0.03
	Male (123)	4.19 ± 1.08	0.12 ± 0.03	0.05 ± 0.03
	Female (152)	3.28 ± 0.77	0.13 ± 0.03	0.06 ± 0.03

Values are presented as mean ± SD. * Significant difference between single and multiple doses group (*p* = 0.000141, 0.000004).

## Data Availability

The raw data supporting the conclusion of this article will be made available by the authors on request.
